# COVID-19 Highlighting Inequalities in Access to Healthcare in England: A Case Study of Ethnic Minority and Migrant Women

**DOI:** 10.1007/s10691-020-09437-z

**Published:** 2020-10-12

**Authors:** Sabrina Germain, Adrienne Yong

**Affiliations:** The City Law School, City, University of London, Northampton Square, Clerkenwell, London, EC1V 0HB UK

**Keywords:** COVID-19, Ethnic minority women, Migrant women, Access to healthcare, Inequality

## Abstract

Our commentary aims to show that the COVID-19 pandemic has amplified existing barriers to healthcare in England for ethnic minority and migrant women. We expose how the pandemic has affected the allocation of healthcare resources leading to the prioritisation of COVID-19 patients and suspending the equal access to healthcare services approach. We argue that we must look beyond this disruption in provision by examining existing barriers to access that have been amplified by the pandemic in order to understand the poorer health outcomes for women in ethnic minority and migrant communities. The reflection focuses on racialised medical perceptions, gendered cultural norms including information barriers and stigma, and specific legal barriers.

## Introduction

As Naqvi and Russell’s recent Editorial (2020) in *Feminist Legal Studies* sharply highlights, it only took six months of a global pandemic to bring to light structural inequalities that have existed for decades. The focus on the higher mortality rate from the virus for men worldwide somewhat eclipses the widening inequalities of gender and race (Flood et al. 2020, 6). Research has shown that ethnic minority and migrant women^1^ are disproportionately affected by existing barriers to access to healthcare (Mirza and Sheridan 2003; Jayaweera 2018). These barriers have been generated and embedded by a system that overlooks the intersectional barriers raised by being an ethnic minority and/or a migrant^2^ woman (Mirza 1997). Our commentary aims to show that the COVID-19 pandemic has amplified these existing barriers in England for ethnic minority and migrant women. We expose how the pandemic has affected the allocation of healthcare resources in England, leading to the prioritisation of COVID-19 patients and suspending the equal access to healthcare services approach. We then explore the poorer health outcomes for women in ethnic minority and migrant communities that the disruption in provision cannot solely account for by looking at the underlying barriers to access that have been amplified by the pandemic. The reflection focuses on racialised medical perceptions, gendered cultural norms including information barriers and stigma, and specific legal barriers.

## COVID-19 Highlighting Unequal Access to Healthcare Services

 The COVID-19 pandemic marks an unprecedented event in healthcare policy. Since the inception of the NHS, the principle of equal access to healthcare services for all has been at the heart of the system, and for the past 70 years, waves of healthcare reforms have aimed to uphold aspects of this foundational approach using different policy strategies (Germain 2019). However, as the number of COVID-19 cases grew rapidly in England, the government implemented healthcare policy prioritising COVID-19 patients to ration resources (NICE 2020), thereby suspending an equal access approach for the delivery of healthcare services for non-COVID patients (Germain 2020).

Had healthcare services kept in line with the traditional egalitarian approach, services would have had to be delivered equally to all patients with similar healthcare needs. Patients would have been treated alike, regardless of whether or not they had contracted the virus. Unfortunately, the reality of the pandemic did not allow for the system to spread its resources in order to preserve equality in access for all. This translated into having some of the routine care for non-COVID patients halted and individuals not directly affected by the virus no longer equally accessing healthcare services based on their needs (BMA 2020).

The National Institute for Health and Care Excellence (NICE) also introduced COVID-19 rapid guidelines that defined criteria for the admission of patients in critical care in March 2020 (NICE 2020). Emergency measures were put in place to maximise health outcomes, favouring individuals with greater chances of survival and introducing measures to rank patients in need of treatment (Shaw et al. 2020). Chronically ill patients were most affected by these measures, especially individuals living with diabetes or cancer that saw their treatment delayed or even cancelled (Extance 2020). These emergency measures have proven to have more permanent consequences in the aftermath of the first peak of infections because of the severe backlog in the system (Neville 2020).

A disproportionately higher number of patients in ethnic minority groups also died during this period (Chaturvedi 2020), making for 15.5 percent of all deaths by June 2020 (Butcher and Massey 2020). The number of deaths recorded during the peak of the pandemic of Central and West African individuals born outside the UK and individuals from the former Eastern bloc of EU Member States was also higher compared to 2014–2018 (PHE 2020, 55).^3^ Ethnic minority women have also made up 55 percent of the pregnant patients admitted with COVID-19, putting them at a higher risk of severe complications (Charity So White 2020). Higher fertility rates in migrant women means that they would have seen their healthcare risk increased during this period too, though no official data exists on this yet (Latif 2014, 1). Our argument is that the suspension of equality in access to care triggered by the emergency measures alone cannot explain these figures. Even though healthcare services were facing unprecedented demand, there were already existing barriers to access the NHS that contribute to poorer health outcomes for ethnic minority and migrant women.

## Barriers to Accessing Healthcare for Ethnic Minority and Migrant Women in Light of COVID-19

Media coverage and government inquiry have focused on the disproportionate impact of the virus on the ethnic minority community without paying any closer attention to how the pandemic has affected men and women differently, apart from the first hand observation that men had recorded more fatalities than women (PHE 2020, 10). There are also no specific policies or guidance on healthcare for vulnerable migrant women (Jayaweera 2018, 277) adding to the difficulties in determining the pandemic’s full impact on migrant women.^4^ However, by critiquing barriers to accessing healthcare from a feminist standpoint, we show that there are specific barriers for women aggravated by their race and cultural associations with race, or having migrant status and being subject to hostile immigration law. We argue that these existing barriers to accessing services have been reinforced, and in some situations deepened, by the pandemic (Lindsay 2020).

### Racialised Medical Perceptions

The prevalence of comorbidities in individuals contracting the virus have been associated with a higher risk of death from COVID-19. However, discussions around the comorbidities affecting the ethnic minority community during and prior to the pandemic have often been tinted with racialised medical perceptions. Patients in the ethnic minority community are not genetically or inherently more prone to develop underlying health conditions, but common socio-economic factors associated with race inequalities have actually led these communities to being more susceptible to these diseases (Roberts 2008). For example, the fact that individuals with South Asian and Afro-Caribbean backgrounds are more susceptible to diabetes or coronary heart diseases and developing a serious and long-term condition thus makes them more vulnerable to the severe effects of COVID-19 (PHE 2020, 40).

However, it is important to note that racialised medical perceptions can impact the judgment of medical professionals and biases are potentially heightened in critical situations where they have to act urgently and instinctively (Grey et al. 2013, 150), such as during a pandemic. There is evidence that medical training received by healthcare professionals perpetuates unconscious biases against marginalised groups, prejudicing future diagnoses of illnesses as well as courses of treatment (Roberts 2008). Ethnic minority and migrant women are more vulnerable to these perceptions because of prejudices and racist beliefs, such as having higher pain thresholds or a greater ability to cope with illness (Laville 2020). They are often also undermined when voicing their healthcare needs.

Even prior to the pandemic, ethnic minority and migrant women had a more restricted access to specialised treatments than white women, notably in the area of maternal care with an increased difficulty in accessing pain relief and a disproportionately higher incidence of mortality during child labour for ethnic minority women (Saini 2020). A lack of appropriate antenatal care could also have knock-on effects specifically on the health of migrant women’s families, increasing migrant children’s likelihood to develop comorbidities themselves (Shortall et al. 2015, 4). It may be that this played out during the pandemic and translated into higher mortality rates in deprived areas (Caul 2020, 2). Ethnic minority and migrant patients have generally been under-represented in medical research and women are also less predominantly the subject of scientific studies making conditions affecting ethnic minority and migrant women least likely to be addressed by medicine.^5^

The suspension of routine healthcare checks and treatments in areas outside intensive and critical care services during the pandemic also had a disproportionate impact on ethnic minority and migrant women. Already, in non-pandemic times, black women were more likely to develop depression and anxiety disorders, but less likely to receive mental health treatment and psychological therapies (Cabinet Office 2018). The stress associated with the risk of contracting the virus and caring for loved ones in lockdown have had an increasingly negative impact on the mental health of many individuals. Ethnic minority and migrant women are in need of greater support during this time of crisis, and the unconscious medical bias and underlying racialisation of their mental health make it less likely for them to be provided with access to adequate mental health services (Latif 2014, 5–7).

### Cultural Barriers Related to Gender

Barriers to accessing healthcare specific to women are not always physical barriers, but rather cultural barriers associated with gender that discourage ethnic minority and migrant women from seeking access to care (Mirza and Sheridan 2003). Specific to the pandemic, this runs counter to the government’s approach in tackling this communicable disease which relies entirely on public cooperation to identify those who have been in contact with the virus (Department of Health and Social Care 2020). Indeed, it is the cultural aspects of these barriers that are not well understood or taken into account in the architecture of the healthcare system. We identify three categories: information, language and communication barriers, gendered cultural norms and stigma associated with seeking care.

Information barriers relating to healthcare mostly concern a lack of information or a lack of adequate communication that impair one’s ability to access services. Language and communication barriers have been an obstacle even before the pandemic, and there is a correlation between ethnicity and deprivation which would potentially exacerbate this.^6^ Barriers arise out of a general lack of confidence in asking questions about seeking care, or being unaware of their right to language support and equality services to help communicate (Health Watch Surrey 2015, 4). Issues for ethnic minority communities include “accessing culturally appropriate services, including lack of cultural understanding, communication issues, and where and how to seek help.” (Grey et al. 2013, 146) Other difficulties cited are the ability to schedule appointments or an unwillingness to ask for help to attend medical appointments, which “frames how [women] were seen in the healthcare system—as a problem.” (Mirza and Sheridan 2003, iv).

We argue that simply stating that all COVID-19 patients can access treatment equally does not remove these pre-existing barriers to access for ethnic minority and migrant women. Specific to the information barrier, the virus’ novelty required new guidance, laws and policies to be rolled out with alarming speed. These new rules needed to be understood, followed and applied appropriately in order to satisfactorily control the virus.^7^ Measures to prevent the spread of the infection, such as the lockdown, government messaging on not overburdening the NHS and fear of catching the virus in hospital settings, would not only be a physical barrier to accessing care, but also exacerbated ethnic minority and migrant women’s health illiteracy (Jayaweera 2014, 5).

These marginalised groups have been forced to develop ‘active strategies and practices which helped them in their struggle to survive.’ This included avoiding ‘statutory and mainstream services and officials where possible.’ (Mirza and Sheridan 2003, iv) However, enforced isolation from the lockdown would have made reliance on their own networks, community and advice more difficult, especially as ‘evidence shows that marginalised ethnic groups have worse internet access,’ (Charity So White 2020) which the pandemic has forced society to increase reliance upon. On the whole, ‘[r]igid and inflexible health care delivery resulted in services excluding the very women they were aimed at helping.’ (Mirza & Sheridan 2003, iii).

### Legal Barriers

Migrant women are more likely to face legal barriers in accessing healthcare because of their migrant status, a result of the reforms made in the Immigration Act 2014. Importantly, effects that migrant women face in this context may intersect with any ethnic minority characteristics they possess, but white migrant women also face discrimination, most prevalent recently against Eastern Europeans in light of Brexit (Rzepnikowska 2019). Noting that ‘migration factors, interacting with ethnicity and socio-demographic factors, may mean that migrants have different needs and meet with different barriers compared to minority ethnic groups’, (Jayaweera 2010, 2) the immigration healthcare surcharge and healthcare policies stemming from the hostile environment are the two main areas of concern regarding barriers to accessing healthcare for migrant women.

Charges are levied against non-nationals seeking healthcare treatment in the UK in three different ways: first, the charges levied on those who are not “ordinarily resident” in the UK;^8^ second, charges for receiving certain types of treatment, such as follow up care after being treated for (free) emergency or immediately necessary care and any secondary (hospital-based or specialist) care; third, the immigration healthcare surcharge payable alongside visa applications. We note that all three scenarios disproportionately impact those on low incomes, which ultimately affects women more because of the high numbers of women in low income jobs, especially because of the numbers of single mothers supporting children. In fact, “[s]ingle mothers, those with precarious employment without social security, and women in frontline caring, domestic and health work, are worse off” (Hankivsky and Kapilashrami 2020).

Since COVID-19 is a novel communicable disease that is more fatal to individuals with comorbidities, we argue that higher fees^9^ may mean that greater numbers of migrants may not have accessed care in the past, putting them at future risk of falling into a high health risk category (Bowsher et al. 2015, 853). The fact that the pandemic preys on those with underlying health conditions shines a spotlight on how existing immigration laws may have contributed to embedding structural inequalities which have played out to the detriment of migrant women today.

The hostile environment policy has also affected access to healthcare for migrants (Kirkup and Winnett 2012). It has been criticised for “medicalising what may be largely social problems.” (Taylor 2009, 769) The law requires NHS trusts to check identification before allowing access to care.^10^ We argue that having to prove identity or immigration status disproportionately impacts women because they are more likely to have precarious or dependent immigration status, having migrated with a male partner as a family dependent (Jayaweera 2018, 274), and likely to have the primary caring responsibilities and atypical work, if any at all. This dependency often becomes the basis of much of their rights in the host territory.

As such, dependency related to immigration status particularly impacts women who are subjects of domestic violence. A migrant woman who relies on the cooperation of an abusive partner faces additional difficulties in accessing identification documents if their abusive partner controls all aspects of their lives, including their whereabouts. This would prevent them from accessing care even if they are eligible (Home Affairs Committee 2017–19). Secondly, and more structurally, if their immigration status depends on their partners, migrant women risk having to stay in an abusive relationship simply to retain a regularised status. The effect of these policies on health is that migrant women often do not seek care until it is too late, thereby adding to their overall poor health and having comorbidities that put them at greater risk of death from COVID-19.

Despite government reassurances that no charges would be levied for treatment of the virus, we note that the policy has not eliminated underlying discrimination against migrant women, whether it be on the basis of race or nationality. It has been argued that “[a]n effective response to the current pandemic requires solidarity among all members of society instead of insistent line drawing between citizens and migrants who are similarly situated.” (Chen 2020, 407) However, the urgency required to control the spread of the pandemic in the UK at the outset meant that little attention was given in the way of addressing the structural inequalities created by legislation. This has led to a disproportionate impact of COVID-19 on ethnic minority and migrant women.

## Conclusion

Existing underlying barriers to accessing healthcare provides some explanation for increased susceptibility to serious negative health outcomes for ethnic minority and migrant women. Our commentary sought to explain that barriers to accessing care have been exacerbated by the COVID-19 pandemic for these marginalised groups of women. These include barriers stemming from racialised medical perceptions, cultural barriers related to gender including information barriers and stigma, and legal barriers stemming from immigration law. Treating women as a homogenous group disregards the individual needs of ethnic minorities and migrants, as doing so embeds structural inequalities that have been highlighted as a result of the pandemic (Platt and Warwick 2020). Our aim was to demonstrate that these structural inequalities in accessing healthcare put ethnic minority and migrant women at greater risk of poorer health outcomes and it appears that this has only worsened because of the COVID-19 pandemic.

## Footnotes

1 In the context of this article, the group of “women” is limited to cisgendered women, equally, the group of “men” is limited to cisgendered men.

2 This commentary will not discuss asylum seekers or undocumented migrants as this category of migrants has been discussed extensively and presents a different set of problems outside the scope of this paper.

3 The former Eastern bloc includes Polish, Bulgarian and Romanian nationals who have notoriously been discriminated against especially since the Brexit vote, and who are also most likely to be in low-income low-skilled positions.

4 Any intersectional discrimination becomes more prevalent because of the lack of information on the health of migrants in general as compared to individuals of different races, ethnic minorities, or genders especially specific to the COVID-19. Migrant women of an ethnic minority background may be disproportionately impacted but currently equality legislation does not adequately address this intersectionality, see Atrey (2019).

5 See Laville 2020. The passing of 36-year-old Kayla Williams illustrates the tragic consequences of racialised medical perceptions. Despite her and her husband’s plea to be taken into hospital for worsening COVID-19 symptoms, paramedics denied their requests as they considered she was not a priority patient.

6 In England, most deprived areas had more than double the mortality rate than least deprived areas for COVID-19. See Caul (2020, 14) and White and Nafilyan (2020) where ‘a recent report also confirms that ‘BAME are also more likely to live in more deprived area than those of White ethnicity.’ There is an information gap for migrant populations due to the difficulty in gathering statistics in particular for EU nationals whose free movement rights in EU law do not require them to register per se and thus no conclusive comments can be made about deprivation and migrant populations.

7 Coronavirus Act 2020; The Health Protection (Coronavirus, Restrictions) (England) Regulations 2020; The Health Protection (Coronavirus, Restrictions) (No. 2) (England) Regulations 2020.

8 Immigration Act 2014, s39(1).

9 Immigration Act 2014, s38.

10 National Health Service (Charges to Overseas Visitors) Regulations 2015, as amended.
